# Predicting MCI Status From Multimodal Language Data Using Cascaded Classifiers

**DOI:** 10.3389/fnagi.2019.00205

**Published:** 2019-08-02

**Authors:** Kathleen C. Fraser, Kristina Lundholm Fors, Marie Eckerström, Fredrik Öhman, Dimitrios Kokkinakis

**Affiliations:** ^1^Digital Technologies Research Centre, National Research Council Canada, Ottawa, ON, Canada; ^2^Department of Swedish, University of Gothenburg, Gothenburg, Sweden; ^3^Institute of Neuroscience and Physiology, Sahlgrenska Academy, Gothenburg, Sweden; ^4^Wallenberg Centre for Molecular and Translational Medicine, University of Gothenburg, Gothenburg, Sweden

**Keywords:** mild cognitive impairment, language, speech, eye-tracking, machine learning, multimodal, early detection

## Abstract

Recent work has indicated the potential utility of automated language analysis for the detection of mild cognitive impairment (MCI). Most studies combining language processing and machine learning for the prediction of MCI focus on a single language task; here, we consider a cascaded approach to combine data from multiple language tasks. A cohort of 26 MCI participants and 29 healthy controls completed three language tasks: picture description, reading silently, and reading aloud. Information from each task is captured through different modes (audio, text, eye-tracking, and comprehension questions). Features are extracted from each mode, and used to train a series of cascaded classifiers which output predictions at the level of features, modes, tasks, and finally at the overall session level. The best classification result is achieved through combining the data at the task level (AUC = 0.88, accuracy = 0.83). This outperforms a classifier trained on neuropsychological test scores (AUC = 0.75, accuracy = 0.65) as well as the “early fusion” approach to multimodal classification (AUC = 0.79, accuracy = 0.70). By combining the predictions from the multimodal language classifier and the neuropsychological classifier, this result can be further improved to AUC = 0.90 and accuracy = 0.84. In a correlation analysis, language classifier predictions are found to be moderately correlated (ρ = 0.42) with participant scores on the Rey Auditory Verbal Learning Test (RAVLT). The cascaded approach for multimodal classification improves both system performance and interpretability. This modular architecture can be easily generalized to incorporate different types of classifiers as well as other heterogeneous sources of data (imaging, metabolic, etc.).

## 1. Introduction

Mild cognitive impairment (MCI) describes a level of cognitive decline characterized by a clinically observable deficit in at least one cognitive domain, but which does not interfere significantly with a person's ability to complete the activities of daily living (Gauthier et al., 2006; Reisberg and Gauthier, 2008). While not everyone with MCI will go on to develop dementia, they are at a greater risk of doing so than the general population (Ritchie and Touchon, 2000). For this reason, it is important to identify these early stages of cognitive decline. There is also increasing evidence that pharmaceutical intervention may be most effective at the milder stages of dementia, before massive neuropathology has occurred (Posner et al., 2017). Identifying individuals in the early stages of dementia is important for clinical trials to test such a hypothesis.

Language tasks have been shown to be sensitive to subtle cognitive deficits (Taler and Phillips, 2008). Due to the time-consuming nature of traditional language analysis, there has been growing interest in the potential application of machine learning technology to help detect cognitive impairment from speech samples. For example, Roark et al. (2011) employed machine learning to distinguish participants with MCI and healthy controls on the basis of an automated analysis of the Wechsler Logical Memory test. Tóth et al. (2015) used support vector machine and random forest algorithms to classify MCI and control participants based on acoustic features derived from a sequence of three narrative speech samples; follow-up work by Gosztolya et al. (2019) additionally incorporated linguistic features, and found that semantic measures and features relating to memory problems were most effective in characterizing the groups. Mirheidari et al. (2017) used automated conversation analysis in the differential diagnosis of participants with mild cognitive difficulties due to neurodegenerative disorders and those with functional memory disorders, finding both non-verbal and semantic cues to be highly predictive. König et al. (2018a) presented an automated method for analyzing the semantic verbal fluency task, and showed that the extracted features could be used to train a machine learning classifier to distinguish between participants with MCI, dementia, and healthy controls. The availability of automated tools for natural language processing, speech analysis, and eye-tracking make it possible to conduct detailed assessment of language production and reception, with minimal need for manual annotation.

In this paper, we propose a multimodal approach to detecting MCI, by combining information from three different language-based tasks (one speech production task and two reading tasks), each of which is observed via at least two different modalities, or modes (audio, text, eye-tracking, and comprehension questions). By integrating information from these different sources, we aim to more accurately distinguish between people with MCI and healthy controls. Additionally, we investigate the predictions made by the classifier at the level of individual modes and tasks, and compare them with standardized neuropsychological test scores.

## 2. Related Work

The discovery of non-invasive biomarkers to detect early stages of cognitive decline in Alzheimer's disease (AD) and related dementias is a significant challenge, and conventional neuropsychological tests may not be sensitive to some of the earliest changes (Drummond et al., 2015; Beltrami et al., 2018). One potential alternative to conventional cognitive testing is the analysis of naturalistic language use, which can be less stressful (König et al., 2015), more easily repeatable (Forbes-McKay et al., 2013), and a better predictor of actual functional ability (Sajjadi et al., 2012). We briefly review the relevant findings with respect to language production and reception in MCI and early-stage AD.

### 2.1. Narrative Speech Production in MCI

Spontaneous, connected speech may be affected in the earliest stages of cognitive decline, as speech production involves the coordination of multiple cognitive domains, including semantic memory, working memory, attention, and executive processes (Mueller et al., 2018), activating numerous areas on both sides of the brain (Silbert et al., 2014). We summarize the previous work examining language and speech in MCI, as well as any reported correlations with cognitive test scores.

The sensitivity of narrative speech analysis to MCI may depend to some extent on the nature of the production task, as different tasks impose different sets of constraints (Boschi et al., 2017). Picture description tasks are the most relevant to our protocol. Cuetos et al. (2007) used the Cookie Theft picture description task from the Boston Diagnostic Aphasia Examination (BDAE) (Goodglass et al., 1983) to elicit speech samples from asymptomatic, middle-aged participants with and without the E280A mutation (which inevitably leads to AD). They found a significant reduction in information content in the carrier group. Ahmed et al. (2013) also analyzed Cooke Theft picture descriptions, and reported deficits in various aspects of connected speech in 15 MCI participants who later went on to develop AD. Impairments were observed in speech production and fluency, as well as syntactic complexity and semantic content. Mueller et al. (2017) analyzed Cookie Theft speech samples from 264 English-speaking participants at two time points, and found that individuals with early MCI (*n* = 64) declined faster than healthy controls on measures of semantic content and speech fluency. Measures of lexical diversity and syntactic complexity did not differ significantly between the groups. Drummond et al. (2015) reported an increased production of repetitions and irrelevant details from MCI participants (*n* = 22) on a task that involved constructing a story from a series of images. However, other work has found no significant differences between MCI and control participants on either verbal (Bschor et al., 2001) or written (Tsantali et al., 2013) Cookie Theft narratives. Forbes-McKay and Venneri (2005) found that while picture description tasks in general can be used to discriminate pathological decline, highly complex images are more sensitive to the earliest stages of decline.

Other work has specifically examined the acoustic properties of speech in MCI and dementia. Temporal and prosodic changes in connected speech are well-documented in AD, including decreased articulation rate and speech tempo, as well as increased hesitation ratio (Hoffmann et al., 2010), reduced verbal rate and phonation rate, and increased pause rate (Lee et al., 2011), and increased number of pauses outside syntactic boundaries (Gayraud et al., 2011). Spectrographic properties, such as number of periods of voice, number of voice breaks, shimmer, and noise-to-harmonics ratio have also been shown to exhibit changes in AD (Meilán et al., 2014). There is evidence that these changes might begin very early in the disease progression, including in the prodromal or MCI stages (Tóth et al., 2015; Alhanai et al., 2017; König et al., 2018b).

Some correlations between characteristics of narrative speech and neuropsychological test scores have been reported for AD (Ash et al., 2007; Kavé and Goral, 2016; Kavé and Dassa, 2018), however, fewer studies have examined possible correlations in the MCI stage. Tsantali et al. (2013) found a significant correlation between performance on an oral picture description task and the Mini-Mental State Examination (MMSE) (Folstein et al., 1975), in a population of 119 Greek participants with amnestic MCI, mild AD, and no impairment. However, MMSE was more highly correlated with other language tasks, including reading, writing, sentence repetition, and verbal fluency.

Mueller et al. (2017) also examined the correlations between measures of narrative speech and standardized neuropsychological test scores, and found only weak correlations: e.g., the correlations between the semantic factor and the Boston Naming Test (Kaplan et al., 2001) and animal fluency task (Schiller, 1947) were positive but not statistically significant. As the authors point out, this may be due to the fact that characteristics of “empty” spontaneous speech, such as an increased production of pronouns, could reflect working memory problems rather than purely semantic impairments (Almor et al., 1999).

To summarize, while the findings with respect to narrative speech production in MCI are somewhat mixed, on our Cookie Theft picture description task we expect the MCI group to show a reduction in semantic content and reduced speech fluency, including a slower rate of speech and increased pausing. Performance on the picture description task may be correlated with scores on the Boston naming test and MMSE score.

### 2.2. Reading in MCI

Reading ability can be assessed in a variety of ways; for example, through reading comprehension, analysis of speech characteristics while reading aloud, and the recording of eye-movements. We summarize the results with respect to MCI along each of these dimensions.

Segkouli et al. (2016) found that when MCI participants were given a paragraph to read and associated questions to answer, they had fewer correct responses and longer time to complete the task, relative to healthy controls. Tsantali et al. (2013) found a strong correlation between MMSE score and the ability to read and comprehend phrases and paragraphs, in participants with amnestic MCI and mild AD. When comparing to healthy controls, they found that reading comprehension was one of the earliest language abilities to be affected in MCI. In a related task, Hudon et al. (2006) examined 14 AD, 14 MCI, and 22 control participants on a text memory task, and found that both MCI and AD participants were impaired on the recollection of detail information and recalling the general meaning of the text. Chapman et al. (2002) reported a similar result, and a comparison with the control and AD groups suggested that detail-level processing is affected earlier in the disease progression. Results, such as this are generally attributed to impairments in episodic memory and a declining ability to encode new information, which can be evident from the early stages of cognitive decline (Belleville et al., 2008).

Further evidence for this hypothesis is given by Schmitter-Edgecombe and Creamer (2010), who employed a “think-aloud” protocol to examine the reading strategies of 23 MCI participants and 23 controls during a text comprehension task. This methodology revealed that MCI participants made proportionally fewer explanatory inferences, which link knowledge from earlier in the text with the current sentence to form causal connections and promote comprehension and understanding. The authors suggest that this could indicate difficulties accessing and applying narrative information stored in episodic memory. They administered a series of true-false questions after each text, and found that MCI participants tended to answer fewer questions correctly, and that comprehension accuracy in the MCI group was correlated with the Rey Auditory Verbal Learning Test (RAVLT) of word learning, as well as RAVLT immediate and delayed recall.

Recent work has used eye-tracking technology to examine reading processes in greater detail. For example, Fernández et al. (2013) recorded the eye movements of 20 people with mild AD and 20 matched controls while they read sentences. They found that the AD patients had an increased number of fixations, regressions, and skipped words, and a decreased number of words with only one fixation, relative to controls. In related work, Fernández et al. (2014) found that participants with mild AD showed an increase in gaze duration. Lueck et al. (2000) similarly reported more irregular eye movements while reading in their mild-moderate AD group (*n* = 14), as well as increased regressions and longer fixation times. Biondi et al. (2017) used eye-tracking data in a deep learning model to distinguish between AD patients and control participants while reading sentences and proverbs. Previous work from our group examined a similar set of features, extending this finding to MCI (Fraser et al., 2017). More generally, Beltrán et al. (2018) propose that the analysis of eye movements (in reading, as well as other paradigms) could support the early diagnosis of AD, and Pereira et al. (2014) suggest that eye movements may be able to predict the conversion from MCI to AD, as eye-movements can be sensitive to subtle changes in memory, visual, and executive processes.

When texts are read aloud, the speech can also be analyzed from an acoustic perspective, in a similar manner to spontaneous speech. De Looze et al. (2018) found that participants with MCI and mild AD generally read slower, with shorter speech chunks relative to controls, and a greater number of pauses and dysfluencies. Segkouli et al. (2016) also observed a reduction in speech rate, reporting a significant positive correlation between the time taken to complete the paragraph reading comprehension task, and the time taken to complete a variety of neuropsychological tests.

Other work has reported increased difficulty in reading words with irregular grapheme-to-morpheme correspondence (i.e., surface dyslexia) in AD (Patterson et al., 1994), although this finding is not universal (Lambon Ralph et al., 1995). A longitudinal study of AD participants concluded that these kinds of surface reading impairments are only significantly correlated with disease severity at the later stages of the disease (Fromm et al., 1991).

In our study, then, we expect that MCI participants will not have difficulty producing the words associated with the texts, but they may read slower and produce more pauses and dysfluencies. MCI participants are expected to answer fewer comprehension questions correctly, as declines in working and episodic memory affect their ability to integrate and retain information from the texts. Their eye movements may show similarities to those of mild AD patients, with an increase in fixations, regressions, and skipped words, and longer gaze duration, although possibly to a lesser extent than has been reported in AD.

### 2.3. Multimodal Machine Learning for MCI Detection

The essential challenge of multimodal learning is to combine information from different sources (i.e., modalities, or modes) to improve performance on some final task, where those information sources may be complementary, redundant, or even contradictory. Traditionally, approaches to multimodal learning have been broadly separated into the two categories of early (or feature-level) fusion and late (or decision-level) fusion, although hybrid approaches also exist (Baltrusaitis et al., 2018). In early fusion, features extracted from different modes are concatenated into a single feature vector, and used to train a classifier. One advantage to this approach is that, depending on the classifier, it can be possible to model relationships between features from different modes. In late fusion, a separate classifier is trained for each mode, and the predictions are then combined, often through a process of voting. One advantage of the late fusion approach is that it avoids the high-dimensional feature space resulting from early fusion, which can make it more appropriate for smaller data sets. Late fusion also offers more flexibility, e.g., the ability to use different classification models for each mode (Wu et al., 1999).

Multimodal learning has been applied to a variety of natural language processing (NLP) tasks, including audio-visual speech recognition (Potamianos et al., 2003), emotion and affect recognition (Schuller et al., 2011; Valstar et al., 2013; D'Mello and Kory, 2015), multimedia information retrieval (Atrey et al., 2010), and many others. With respect to dementia detection, multimodal approaches have been most effective in the medical imaging domain, where such methodologies have been used to combine information from various brain imaging technologies (Suk et al., 2014; Thung et al., 2017). For example, work from Beltrachini et al. (2015) and De Marco et al. (2017) has shown that the detection of MCI can be improved when combining features from MRI images with cognitive test scores in a multimodal machine learning classifier, compared to learning from either data source individually.

However, previous NLP work on detecting MCI and dementia has typically focused on language production elicited by a single task, such as picture description (Fraser et al., 2016; Yancheva and Rudzicz, 2016), story recall (Roark et al., 2011; Lehr et al., 2013; Prud'hommeaux and Roark, 2015), conversation (Thomas et al., 2005; Asgari et al., 2017), or tests of semantic verbal fluency (Pakhomov and Hemmy, 2014; Linz et al., 2017). In cases where more than one speech elicitation task has been considered, the approach has typically been to simply concatenate the features in an early fusion paradigm.

For example, Toth et al. (2018) consider three different speech tasks, concatenating speech-based features extracted from each task for a best MCI-vs.-control classification accuracy of 0.75. They do not report the results for each task individually, so it is not possible to say whether one task is more discriminative than the others. In a similar vein, König et al. (2018b) combine features from eight language tasks into a single classifier, and distinguish between MCI and subjective cognitive impairment with an accuracy of 0.86, but include only a qualitative discussion of the relative contributions of each of the tasks to the final prediction. Gosztolya et al. (2019) use a late fusion approach to combine linguistic and acoustic features for MCI detection; however, the data from their three tasks was again merged into a single feature set for each mode, obscuring any differences in predictive power between the tasks.

### 2.4. Hypotheses

Previous work has found that speech, language, eye-movements, and comprehension/recall can all exhibit changes in the early stages of cognitive decline. Furthermore, tasks assessing these abilities have been successfully used to detect MCI using machine learning. However, to our knowledge there has been no previous work combining information from all these various sources, and the few studies in the field which have explored multimodal classification have primarily focused on a single approach to fusing the data sources. Additionally, there has been no prior work attempting to link the predictions generated by a machine learning classifier to standardized neuropsychological testing. Thus, the two questions that we seek to answer in the current study are:

1. **Can we improve the accuracy of detection of MCI by combining information from different modes and tasks, and at what level of analysis is the information best integrated?** Our hypothesis is that combining all the available information will lead to better performance than using any single mode or task.
2. **Do the predictions made by the machine learning classifier correlate with participant scores on standard tests of language and other cognitive abilities?** Our hypotheses are: (a) Neuropsychological tests which are timed will be correlated with predictions based on the speech mode, which also encodes timing information; (b) Neuropsychological tests in the language domain will be correlated with predictions based on the language mode; and (c) Predictions which combine information from all modes and tasks will be correlated with MMSE, which also involves many cognitive domains. Since there is no previous work correlating eye-movements while reading with cognitive test scores, we do not generate a specific hypothesis for this mode, although we do include it in the analysis.

## 3. Methods

We first describe the participants and the details of the language tasks, then define the variables that are extracted from each data mode (audio, text, eye-movements, and comprehension questions). We then present the different machine learning architectures and the details of the classification procedure.

### 3.1. Participants

The participants in this study were recruited from the Gothenburg MCI Study, a longitudinal study investigating early and manifest phases of different dementia disorders in patients seeking medical care at a memory clinic (Wallin et al., 2016). The Gothenburg MCI Study has been approved by the local ethical committee review board (reference number: L091–99, 1999; T479–11, 2011); while the currently described study has been approved with reference number (206–16, 2016). A total of 31 MCI patients and 36 healthy controls (HC) were recruited according to detailed inclusion and exclusion criteria, the details of which can be found in Kokkinakis et al. (2017). Briefly, these criteria specified that all participants should be in the age range 50–79 years, speak Swedish as their first language and as the only language before the age of 5 years, and should have recent neuropsychological test results available as a result of their participation in the Gothenburg MCI Study. Participants were excluded if their cognitive impairment was due to some cause other than neurodegenerative disease (e.g., stroke, brain tumor), if they suffered from depression, substance abuse, serious psychiatric impairment, or neurological disease, if they had a history of reading impairment (e.g., dyslexia), or if they had any uncorrected vision or hearing difficulties. Written, informed consent was obtained from all participants.

In the current analyses, we included only participants who had completed all three of the tasks (Cookie Theft, reading silently, and reading aloud), which led to a final dataset comprising 26 MCI participants and 29 HC participants. Demographic data is given in Table 1.

**Table 1:** Demographic information and neuropsychological test scores, by group (mean and standard deviation).

		**HC (*n* = 29)**	**MCI (*n* = 26)**	**Sig**.
	Age (years)	67.8 (7.7)	70.6 (5.8)	*
	Education (years)	13.3 (3.7)	14.3 (3.6)	n.s.
	Sex (F/M)	21/8	14/12	n.s.
	MMSE (/30)	29.6 (0.6)	28.2 (1.4)	***
Memory/Learning	RAVLT (total)	45.5 (11.1)	37.6 (10.7)	*
	RAVLT (delayed)	9.2 (3.6)	5.8 (3.5)	***
	RAVLT (immediate)	9.5 (3.5)	6.1 (3.1)	***
	RCF (3 min)	18.8 (5.1)	15.8 (6.8)	n.s.
	RCF (20 min)	18.6 (4.4)	14.3 (7.0)	*
	WLM (delayed)	21.9 (8.1)	16.0 (10.5)	*
	WLM (immediate)	25.8 (6.3)	21.3 (7.6)	*
Language	BNT	53.3 (4.6)	50.2 (7.6)	n.s.
	Verbal fluency (F-A-S)	47.2 (11.5)	43.6 (11.1)	n.s.
	Similarities	24.6 (4.7)	24.0 (5.2)	n.s.
	Token test (Part 5)	20.9 (1.4)	20.0 (1.8)	n.s.
Attention	Digit span	13.1 (3.5)	12.4 (2.8)	n.s.
	Digit-symbol	62.9 (12.3)	54.2 (10.8)	**
	TMT A	34.1 (11.9)	39.5 (13.3)	n.s.
	TMT B	79.8 (32.9)	97.8 (49.4)	n.s.
Spatial	Block design	40.6 (9.5)	35.5 (12.2)	n.s.
	RCF (copy)	33.6 (2.4)	32.4 (3.4)	n.s.
	Silhouettes	22.4 (4.2)	19.3 (3.3)	***
Executive	Letter-digit	9.5 (2.3)	8.7 (2.6)	n.s.
	PaSMO	68.2 (21.5)	86.8 (29.1)	*
	Stroop (trial 1)	13.2 (2.4)	14.6 (3.1)	n.s.
	Stroop (trial 2)	17.6 (3.4)	19.4 (5.4)	n.s.
	Stroop (trial 3)	24.1 (6.6)	27.6 (6.6)	*
	Stroop effect	1.8 (0.4)	1.9 (0.5)	n.s.

* indicates p < 0.05, ** indicates p < 0.01, *** indicates p < 0.005.

Through the Gothenburg MCI study, all participants were assessed by a registered nurse, who administered a number of cognitive tests and symptom checklists (Wallin et al., 2016). For control participants (recruited primarily through information sessions at senior citizens' organizations), the assessment ruled out any subjective or objective cognitive impairment. Participants who had been referred to the memory clinic due to memory complaints were assessed for their level of cognitive decline according to the Global Deterioration Scale (GDS) (Auer and Reisberg, 1997). This assessment included the Mini-Mental Status Examination (MMSE), listed in Table 1, as well as the Clinical Dementia Rating (Morris, 1997), Stepwise Comparative Status Analysis (Wallin et al., 1996), and a short form of the executive interview EXIT (Royall et al., 1992). Those participants categorized as GDS stage 3 were included in the MCI group per the original Petersen criteria (Petersen et al., 1999); participants classified at any other stage were excluded from the current analysis. According to standard clinical protocol, no biomarker evidence was considered in the diagnostic criteria.

All participants also completed a comprehensive neuropsychological battery, the results of which are summarized in Table 1. The neuropsychological test battery was administered at the memory clinic by a licensed psychologist or a supervised psychologist in training. The examination was performed in two sessions of 1.5–2 h. The battery covered a range of cognitive areas, including *learning and memory* (Rey Auditory Verbal Learning Test, or RAVLT; Rey Complex Figure, or RCF, recalled after 3 and 20 min; and Weschler Logical Memory subtest, or WLM), *language* (Boston Naming Test, or BNT; verbal fluency for letters F-A-S; Similarities subtest from the Wechsler Adult Intelligence Scale, or WAIS; and the Token Test, part 5), *attention* (WAIS Digit Span test; WAIS Digit-Symbol test; Trail-Making Test, or TMT, forms A and B), *visuo-spatial ability* (WAIS Block Design test; RCF copy; and Silhouettes subtest from the Visual Object and Space Perception Battery), and *executive function* (WAIS Letter-Digit subtest; Parallel Serial Mental Operations, or PaSMO; and Stroop test). Note that none of these tests were used in the diagnostic procedure described previously.

The protocol for the Gothenburg MCI study does not include subtyping of MCI participants, and thus our analysis does not take into account any heterogeneity within our MCI population. However, for the sake of interpretation, we did perform a retrospective analysis of the subtype composition of our sample based on the neuropsychological test scores, following the methodology described in Göthlin et al. (2017). Four MCI subtypes (aMCI-sd, amnestic single domain MCI; aMCI-md, amnestic multi domain MCI; naMCI-sd, non-amnestic single domain MCI; naMCI-md, non-amnestic multi domain MCI) were calculated using neuropsychological test data, with normal scores based on healthy control data and stratified by age group (50–64 and 65–80 years), using a 1.5 standard deviation cut-off for MCI. The subtypes were operationalized as follows: aMCI-sd: at least one memory test score below the cut-off score and all non-memory domain test scores above cut-off; aMCI-md: at least one memory test score and one non-memory domain test score below cut-off; naMCI-sd: at least one non-memory test score below cut-off and all memory test scores above cut-off; naMCI-md: at least two test scores below cut-off in any two non-memory domain tests and all memory test scores above the cut-off score. Specific cut-off scores are listed in Göthlin et al. (2017).

Of the 26 patients that were categorized as MCI using the GDS algorithm, the neuropsych-based subtyping resulted in: 3 patients categorized as aMCI-sd; 9 as aMCI-md; 5 as naMCI-sd: and 3 as naMCI-md. Six patients did not fulfill the neuropsychological test criteria (even though they fulfilled the GDS criteria), and could therefore not be categorized into a subtype using neuropsychological tests. Two of those six had missing neuropsychological data, which could possibly explain why these individuals did not fulfill the neuropsychological MCI criteria. Two aMCI-sd and one naMCI-sd patients also had missing data (not in the memory domain), which makes it uncertain if these were truly “single domain” cases.

### 3.2. Data Collection

In a separate session, the participants completed the three tasks that we consider here. This session took place in a quiet lab environment at the University of Gothenburg. For the two reading tasks, we used an EyeLink 1000 Desktop Mount with monocular eye-tracking and a sampling rate of 1,000 Hz, with a headrest for head stabilization. The participants read two short texts, and after each they answered five comprehension questions. The first text was read silently, while the second was read aloud. In the reading aloud condition, the speech was recorded using a H2n Handy recorder and the audio files were stored in WAV format with a sampling rate of 44.1 kHz and 16-bit resolution.

Both reading texts were taken from the International Reading Speed Texts (IReST), which is a collection of texts that is available in 17 different languages. They are 146 words long in Swedish, and were developed to be used as an evaluation tool for impairments in vision or reading ability (Trauzettel-Klosinski and Dietz, 2012). We chose to present complete paragraphs (rather than individual sentences) to simulate a more natural reading task, requiring the integration and recollection of information from the beginning through to the end of the paragraph.

Areas of interest (AOIs) were defined in the text, with each word labeled as a separate AOI. Eye movements, such as saccades and fixations, are then calculated with respect to the predefined AOIs. Fixations occurring outside the AOIs are not considered in this analysis.

The eye-tracker was calibrated for each participant, and drift-corrected between Trials 1 and 2. However, visual inspection of the data revealed a tendency for downward drift, particularly in the second trial. This was corrected manually, where necessary, to the degree agreed upon by two of the authors (KF and KL).

Participants also described the Cookie Theft picture, which is widely used to elicit narrative speech (Goodglass et al., 1983). The stimulus image shows a boy standing on a stool, trying to reach a cookie jar which sits on a high shelf. A girl stands nearby, and a woman washes dishes at the sink and gazes distractedly out the window. Participants were asked to describe everything they saw in the picture, and to talk for as long as they liked. The narratives were recorded and later manually transcribed by a professional transcriber, according to a detailed protocol developed by the authors.

### 3.3. Feature Extraction: Sessions, Tasks, Modes, and Features

**Figure 1:**
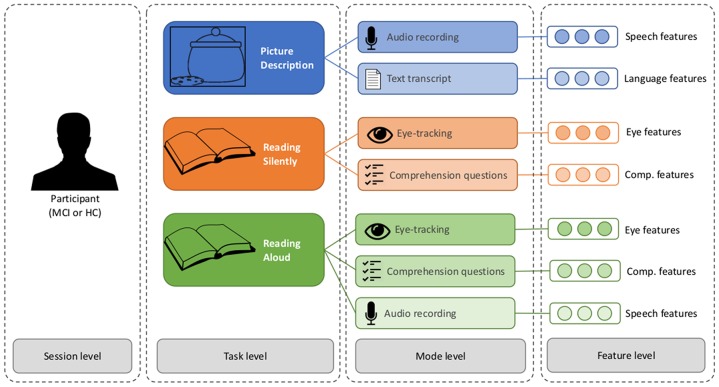
The different levels of analysis in the multimodal dataset.

To avoid confusion, we will refer to the different levels of data analysis using the following terminology (see Figure 1): Each participant takes part in a data-collection *session*. Each session consists of three *tasks*: the picture description task, the silent reading task, and the verbal reading task. Information about each task is captured through different *modes*: for the picture description, we consider the speech signal and the transcript of the words that were produced, when reading silently we consider the eye-movements and the responses to the comprehension questions, and when reading aloud we consider the eye-movements, the comprehension questions, and the speech signal. Each mode is represented by a set of variables, or *features*. The features are motivated by the related work, and described in the following tables: language features are given in Table 2, speech features in Table 3, eye-movement features in Table 4, and comprehension features in Table 5. The feature values for each mode are stored in a feature vector, which constitutes the input the machine learning classifier.

**Table 2:** Language features (26 features).

**Total words** is the total number of words produced (excluding filled pauses, unintelligible words, and false starts) (1 feature).
**Mean length of sentence (MLS)** is the total number of words in the narrative divided by the number of sentences (1 feature).
**Phrase type proportion** is derived from work on rating the fluency of machine translations (Chae and Nenkova, 2009). The phrase type proportion is the total number of words belonging to a given phrase type (here prepositional phrases, noun phrases, and verb groups), divided by the total number of words in the narrative. We additionally extend this feature to apply to clauses; namely main finite clauses, main infinitive clauses, and subordinate clauses (6 features).
**Part-of-speech ratios** are computed for: the ratio of nouns to verbs, the ratio of pronouns to nouns, the ratio of determiners to nouns, and the ratio of open-class words to closed-class words (4 features).
**Proportion of verbs in the present tense** is computed as a proxy for *discourse type* as presented in Drummond et al. (2015), distinguishing between a descriptive style (mostly present tense) vs. narrative style (mostly past tense) (1 feature).
**Median word frequency** is estimated according to the modern Swedish section of the Korp corpus (Borin et al., 2012) (1 feature).
**Type-token ratio (TTR)** is calculated by dividing the number of unique word types by the total number of tokens in the narrative (1 feature).
**Information unit counts** are computed for each of the information unit categories listed in (Croisile et al., 1996); namely, the three *subjects*, eleven *objects*, two *places*, and seven *actions*. These counts are extracted using a keyword-spotting method with some manual correction. The total count for each category is then normalized by the total number of words in the narrative (4 features).
**Content density** and **content efficiency** are computed by counting the total number of information units mentioned (including repetitions) and dividing by the total number of words and the total time, respectively (2 features).
**Propositional density** is calculated by taking the ratio of propositions (verbs, adjectives, adverbs, prepositions, and conjunctions) to total number of words (Mueller et al., 2017) (1 feature).
**Dysfluency marker counts** are computed by counting the number of filled pauses, false starts, and incomplete sentences, each normalized by total number of words. An overall dysfluency index is also computed by summing the counts from the three categories and dividing by the total number of words (4 features).

**Table 3:** Speech features (12 features).

**Syllable count:** Number of syllables produced by the speaker, as estimated using the Praat script of De Jong and Wempe (2009) (1 feature).
**Pause count:** Number of pauses longer than 150 ms (1 feature).
**Duration:** Total duration of the speech sample (1 feature).
**Phonation time:** Total duration of time spent in speech (i.e., excluding silent pauses) (1 feature).
**Proportion of time spent speaking:** Phonation time divided by total duration (1 feature).
**Speech rate:** Number of syllables, divided by total duration (1 feature).
**Average syllable duration (ASD):** Phonation time, divided by number of syllables (1 feature).
**Pauses per syllable:** Number of pauses, divided by total number of syllables (1 feature).
**Pause rate:** Number of pauses, divided by total duration (1 feature).
**Pause duration (mean and s.d.):** The mean and standard deviation of the duration of pauses >150 ms (2 features).
**Maximum pause duration:** Maximum pause length over all pauses in the sample (1 feature).

**Table 4:** Eye-movement features (22 features).

**Saccade amplitude (mean and s.d.):** The mean and standard deviation of the amplitude of the saccades, averaged over all saccades in a trial (2 features).
**Saccade distance (mean and s.d.):** The mean and standard deviation of the number of words covered in a saccade, averaged over all saccades in a trial (2 features).
**Regression amplitude (mean and s.d.):** The mean and standard deviation of the amplitude of the regressions (saccades ending on an AOI that precedes the AOI on which they started), averaged over all regressions in a trial (2 features).
**Regression distance (mean and s.d.):** The mean and standard deviation of the number of words covered in a regression, averaged over all regressions in a trial (2 features).
**Max regression amplitude:** Maximum amplitude of a regressive saccade (1 feature).
**Max regression distance:** Maximum number of words covered in a regressive saccade (1 feature).
**Fixation count:** The total number of fixations in a trial (1 feature).
**First-pass first fixation count:** The total number of first fixations occurring in the first pass of a trial. That is, a first-pass first fixation occurs when it is the first fixation on the given word, and there have been no fixations on any words occurring later in the text (1 feature).
**Later-pass first fixation count:** The total number of first fixations occurring outside the first-pass of a trial. That is, a later-pass first fixation occurs when it is the first fixation on the given word, but there have already been fixations on words occurring later in the text (1 feature).
**Multi-fixation count:** The total number of fixations on a word in the first-pass, excluding the first fixation. That is, a multi-fixation occurs when a word is fixated on multiple times in the run which starts with a first-pass first fixation (1 feature).
**Re-fixation count:** The total number of fixations on a word outside the first pass, excluding the first fixation (1 feature).
**Reading fixation count:** Number of fixations occurring before the first visit to the last AOI in the text (1 feature).
**Re-reading fixation count:** Number of fixations occurring after the last AOI has been visited at least once (1 feature).
**First-pass first fixation duration (mean and s.d.):** The mean and standard deviation of the duration of the first-pass first fixations (2 features).
**Wrap-up gaze duration (mean and s.d.):** The mean and standard deviation of the gaze time on the last word in a sentence, which is typically longer than normal gaze time, to allow extra time for semantic processing and integration (Payne and Stine-Morrow, 2014) (2 features).
**Wrap-up ratio:** Ratio of mean wrap-up gaze time to mean sentence-internal gaze time (1 feature).

**Table 5:** Comprehension features (11 features).

**Average accuracy:** Proportion of questions that were answered correctly (1 feature).
**Question correctness:** For each of the five questions, a binary-valued feature indicating whether the question was answered correctly or incorrectly (5 features).
**Question response time:** For each of the five questions, the time (in ms) taken for the participant to read and respond to the question (5 features).

### 3.4. Classification

**Figure 2:**
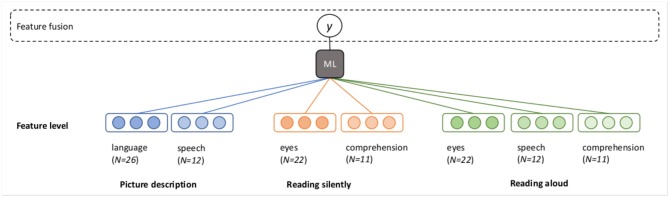
Fusion at the feature level (early fusion).

**Figure 3:**
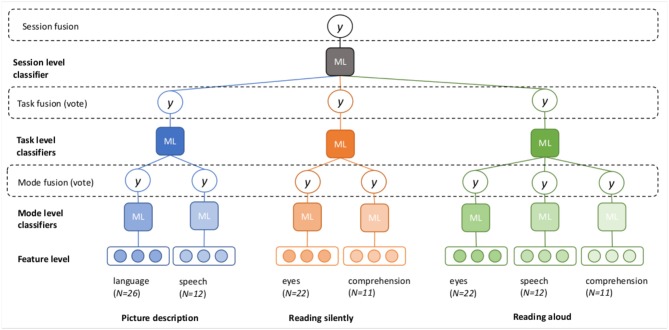
Fusion at the mode, task, and session levels (late fusion).

The goal of the classification experiments is to predict the diagnosis (or “class”) of a participant (MCI or HC), based on the feature vectors. The question at hand is how to best combine the seven feature vectors described in Figure 1. We propose several possible architectures in Figures 2, 3. In these schematics, a box labeled “ML” represents a generic machine learning classifier, which takes a feature vector as input, and outputs a prediction *y*, where *y* is a score between 0 and 1, representing a probability estimate that the feature vector came from a control participant (coded as 0) or MCI participant (coded as 1). The final classification is obtained by thresholding this score (default threshold: 0.5).

In the early fusion paradigm, which we will call *feature fusion*, the feature vectors from each mode for every task are concatenated into a single long vector for each participant, and one classifier is trained to predict the output class (Figure 2).

Three different options for late fusion are shown in Figure 3. In the *mode fusion* configuration, a separate classifier is trained for each mode, for each task. This results in seven different probability scores, which are averaged to produce the final prediction. We can also assess the accuracy of each individual classifier, to observe which modes are the most predictive.

In the *task fusion* configuration, the outputs (probability scores) of the mode-level classifiers are fed into the next layer of task-level classifiers. The task-level classifiers again output probability scores, which are averaged to produce the task-level prediction. We can now evaluate the accuracy of the classification for each task, as well as each mode within a task.

In the final configuration, the outputs from the task-level classifiers are now used to train a final, *session fusion* classifier. In this set-up, probability estimates are available at all levels of analysis.

For this study, we implement each general architecture with both logistic regression (LR) and support vector machine (SVM) classifiers, using Python and scikit-learn (Pedregosa et al., 2011). Our expectation is that if the results are robust, we should see similar results with both types of classifier. Because SVM classifiers do not output probability scores directly, we use Platt's method to generate probability estimates from the decision function, as implemented in the CalibratedClassifierCV method (Platt, 1999). While LR is a linear model, with SVM we allow for non-linear classification by using the radial basis function (RBF) kernel. For both LR and SVM, we use L2 regularization to help prevent over-fitting. Because we want to combine the classifier scores in a meaningful way, we do not optimize the regularization parameter independently across classifiers; rather, we use the default setting of *C* = 1.0 in all cases.

To evaluate classifier performance, our primary metric will be the area under the receiver-operator characteristic curve (henceforth simply AUC), which is commonly used as a measure of discriminative ability for clinical tests. In all cases, we use a leave-pair-out cross-validation strategy, which involves iterating over the dataset such that every possible MCI-HC pair is held out as the test set once, with the remaining samples used as the training set. This alternative to leave-one-out cross-validation has been shown to produce an unbiased estimate for AUC on small data sets (Airola et al., 2009), and has also been used in related work (Roark et al., 2011; Prud'hommeaux and Roark, 2015). We estimate AUC and its standard deviation using Equations (3) and (4) from Roark et al. (2011). For the sake of completeness, we also report accuracy, sensitivity (true positive rate), and specificity (true negative rate).

As a baseline for comparison, we additionally train LR and SVM classifiers using the neuropsychological test scores as features. This allows us to compare the predictive ability of our data with standardized psychometric tests. Since these data are not multimodal, we simply consider the standard classification approach here. Some participants are missing some of the test scores; in these cases we use feature imputation (sklearn.impute.SimpleImputer) to handle the missing values.

## 4. Results

We first present the classification results obtained at each level of the cascaded classifier. We then examine the average probability score assigned to each participant in our cohort, and give an example of how the cascaded approach can be used to provide explanations at various levels of analysis for any given individual. Finally, we report correlations between the classifier predictions and the neuropsychological test scores.

### 4.1. Classification of MCI vs. Controls

**Figure 4:**
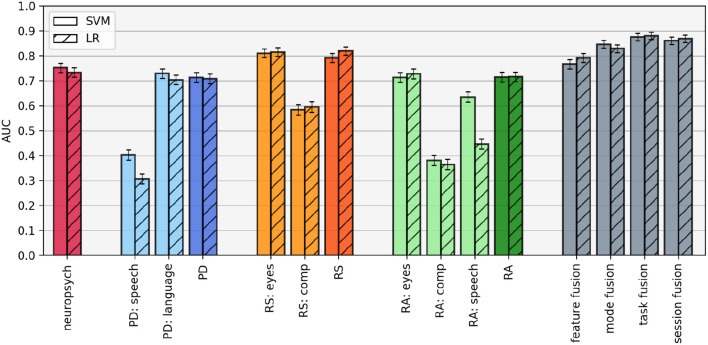
Area under the ROC curve (AUC) for each classification configuration, estimated using leave-pair-out cross-validation. Error bars represent standard deviation over each fold in the cross-validation. PD, picture description; RS, reading silently; RA, reading aloud.

The classification results for each mode and task classifier, as well as the four fusion methods and the neuropsychological test score baseline, are shown in Figure 4. There are several observations to be made:

- **Results are relatively classifier-independent**. While the results are not identical, the same patterns emerge using LR and SVM, as expected. The only obvious discrepancies occur in the speech mode. In both the picture description and reading aloud tasks, the SVM classifier achieves substantially better results than the LR classifier in the speech mode, suggesting that the non-linear kernel confers some benefit here.
- **Within tasks, one mode dominates**. In each of the three tasks, the task-level AUC is essentially equivalent to the best mode-level AUC within that task. Adding additional information from the less-accurate mode(s) does not increase the overall classifier performance on that task. This observation is confirmed by examining the weights assigned to each of the modes in the task-level classification: for LR, on average, the picture description language mode is weighted 45.2 times higher than the speech mode, the silent reading eye-tracking mode is weighted 3.0 times higher than the comprehension mode, and the verbal reading eye-tracking mode is weighted 44.0 times higher than the comprehension mode and 6.6 times higher than the speech mode. The benefit to this phenomenon is that task-level classifiers are not negatively impacted by unreliable information coming from mode-level classifiers with poor predictive ability; the drawback is that there is no boost to performance by combining potentially complementary information.
- **Mode informativeness varies across tasks**. Looking at the mode-level classifiers, we see that the speech signal leads to better performance in the verbal reading task than the picture description task, and both the eye-movements and comprehension questions lead to better results in the silent reading task than the verbal reading task.
- **Within a session, the silent reading task is most predictive**. The picture description and verbal reading tasks result in AUCS of 0.71 and 0.72, respectively (for both classifiers). These values are comparable, but slightly worse, than the baseline classifiers trained on the neuropsychological test scores (LR AUC = 0.73 and SVM AUC = 0.75). However, the silent reading task out-performs both the other tasks and the baseline, with LR AUC = 0.82 and SVM AUC = 0.79.
- **Task fusion leads to the best results**. Both LR and SVM achieve a maximum AUC of 0.88 in the task fusion paradigm, although the error bars in this condition overlap with the session fusion results. Note that the only difference between these two paradigms are that in task fusion, the predictions are combined in an unweighted average, while in session fusion, the predictions from each task are weighted according to the parameters learned by the top-level classifier. In the LR classifier, we can observe that these learned weights are close to 1.0 (i.e., equivalent to task fusion), with the silent reading prediction being weighted on average only 1.2 times more than the picture description and 1.3 times more than the verbal reading prediction.

Accuracy, sensitivity, and specificity scores for each classifier are given in Table 6. The best result for each column is highlighted in boldface. Again, we observe that the best results are obtained by combining the information from the different tasks and modes. The improvement in performance over the neuropsychological test score baseline is even more apparent when considering classification accuracy, where the task fusion paradigm leads to an accuracy of 0.83 in both cases, compared to 0.62 (LR) and 0.65 (SVM) with the standardized test scores.

**Table 6:** Classification results for the baseline classifier trained on neuropsychological test scores, followed by the results at each level of the cascaded classifier.

	**AUC**		**Accuracy**		**Sensitivity**		**Specificity**	
	**LR**	**SVM**	**LR**	**SVM**	**LR**	**SVM**	**LR**	**SVM**
Neuropsych.	0.73	0.75	0.62	0.65	0.53	0.56	0.70	0.74
PD: speech	0.31	0.40	0.46	0.51	0.09	0.15	0.83	0.87
PD: language	0.70	0.73	0.69	0.69	0.52	0.54	0.86	0.84
PD: combined	0.71	0.71	0.67	0.63	0.55	0.55	0.79	0.72
RS: eyes	0.82	0.81	0.72	0.72	0.70	0.66	0.75	0.79
RS: comp	0.60	0.58	0.57	0.58	0.40	0.41	0.73	0.75
RS: combined	0.82	0.79	0.70	0.68	0.68	0.65	0.72	0.72
RA: eyes	0.73	0.71	0.66	0.65	0.64	0.62	0.69	0.68
RA: comp	0.37	0.38	0.48	0.41	0.18	0.06	0.77	0.76
RA: speech	0.45	0.64	0.43	0.56	0.25	0.38	0.62	0.73
RA: combined	0.72	0.72	0.65	0.65	0.63	0.62	0.67	0.67
Feature fusion	0.79	0.77	0.69	0.70	0.74	0.70	0.64	0.71
Mode fusion	0.83	0.85	0.75	0.76	0.56	0.59	**0.93**	**0.93**
Task fusion	**0.88**	**0.88**	**0.83**	**0.83**	0.78	**0.80**	0.89	0.85
Session fusion	0.87	0.86	0.80	0.78	**0.81**	0.79	0.79	0.77

*PD, picture description; RS, reading silently; RA, reading aloud. The best result for each classifier and metric is indicated in boldface*.

The sensitivity and specificity metrics indicate the types of errors made by the classifiers. For example, the comprehension mode classifier of the reading aloud task has a sensitivity of only 0.06 in the SVM case, indicating that almost all MCI participants were mis-classified as controls. The speech mode of the picture description task also suffers from extremely low sensitivity. In contrast, most classifiers have reasonable specificity, with the lowest scores appearing in the verbal reading task, and the highest scores in the mode fusion paradigm.

### 4.2. Prediction on an Individual Level

**Figure 5:**
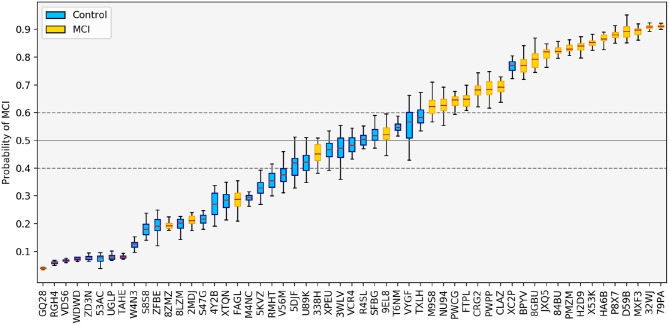
Predictions made by the LR classifier using feature fusion, averaged across all test folds. The solid gray line indicates the default threshold of 0.5. By changing the threshold, we can adjust the sensitivity and specificity of the classification; e.g., if the threshold is increased to 0.6 (upper dashed line), only one control is mis-classified as MCI; if the threshold is decreased to 0.4 (lower dashed line), only four MCI patients are mis-classified.

While it is most common to only report classification results at the group level, it can be informative to examine the predictions on an individual level as well. For each participant, the probability of MCI according to the top-level session fusion LR classifier, averaged across all cross-validation experiments, is shown in Figure 5. At the extreme ends, most participants assigned the highest and lowest probability of MCI are correctly classified. The standard deviation is also smaller, indicating that the classifiers' estimates in those cases are more confident and robust against small changes in the training data. In the middle of the probability range, we see more mis-classifications and bigger errors bars, suggesting greater uncertainty around these predictions. Figure 5 also illustrates how the decision threshold can be shifted up or down to change the sensitivity and specificity of the classifier, depending on the practical application.

Figure 5 also reveals an unexpected error in the classification: the classifier ranks an MCI participant (with ID GQ28) as the least likely to have MCI in the entire dataset (this result is also seen in the SVM case). This error case will be discussed in more detail below.

Considering these false negatives in the context of our retrospective subtyping analysis, we find that the five misclassified participants shown in Figure 5 all come from different groups: GQ28 is classified as naMCI-sd, 8ZMZ was not assigned a subtype according to the neuropsychological test scores, 2MDJ is classified as aMCI-sd, FAGL is classified as aMCI-md, and 338H is classified as naMCI-md. From this we conclude in general that the classifier is learning various patterns of impairment and is not performing especially poorly on any specific subtype.

### 4.3. Multi-Level Interpretation

When designing machine learning systems in a healthcare context, high accuracy is typically a necessary but not sufficient condition for an acceptable system; generally some level of interpretability is also required. While the notion of interpretability can be vague, and its evaluation requires careful consideration (see Doshi-Velez and Kim, 2017 for a discussion), Lipton (2016) suggests that two properties of interpretable models are *transparency* and *post-hoc interpretability*. We briefly and qualitatively discuss these properties in relation to the models considered here.

On the surface, the classifier outputs either a 0 (HC) or 1 (MCI) label for each participant. However, a benefit of the cascaded approach is that individual-level predictions, such as those shown in Figure 5, are available at every level. In that sense, we are allowed to peer into the “black box” at various levels of the prediction, allowing for *post-hoc* explanations of how the classifier arrived at its final decision.

For our first example, we consider the output of the LR classifier for participant 84BU, who was correctly identified as having MCI. In addition to the binary 0/1 output, the system might prepare an explanatory report, such as the following: “This patient has an overall probability of 0.82 of having MCI. While performance on the picture description was normal [*p*(MCI) = 0.27], both reading tasks suggest a high probability of impairment, with *p*(MCI) = 0.86 and *p*(MCI) = 0.89 for reading silently and aloud, respectively. Specifically, the eye-movements during the silent reading task suggest a possible impairment [*p*(MCI) = 0.73].” If desired, the explanation could even continue on to the level of the individual features at each mode. Explanations of this nature increase the transparency of the decision process.

In fact, the classifier trained on neuropsychological test scores also classified this participant correctly; while the MMSE score was 30/30, the detailed neuropsychological test battery revealed a significant memory impairment.

We can take a similar approach to examine the error case mentioned in the previous section. Participant GQ28 was assigned a very low probability of MCI, despite actually having that diagnosis. Every classifier in the cascaded architecture assigned a probability of MCI <0.5, except for the comprehension-level classifier in the silent reading task, which assigned a score of *p*(MCI) = 0.67.

Examining the features, the only features which indicate impairment are the reaction times, which are much slower for this participant than in the control group. Interestingly, the neuropsychology classifier also mis-classifies this participant, and examining the test scores, it appears that the only tests on which this participant scores abnormally (<1.5 standard deviations below the mean) are the PaSMO and Token Test; on many others the scores are in fact better than the control average. In particular, this participant scores highly on all the memory tests. This suggests a limitation of both the small dataset and the classification scheme: if an impairment is small and atypical (relative to the rest of the training set), then the signal may get “out-voted” in the fusion process.

### 4.4. Correlation Between Classifier Predictions and Neuropsychological Test Scores

In the previous section, we examined the classifier outputs for two MCI participants (one correctly classified and one incorrectly classified), and on a surface level it appeared that there may be some link between the classifier predictions and neuropsychological testing performance. Such a relationship could potentially be used to better ground the predictions in clinical knowledge. In this section, we examine the correlations between the neuropsychological test scores and the classifier predictions, at the level of modes, tasks, and sessions.

**Figure 6:**
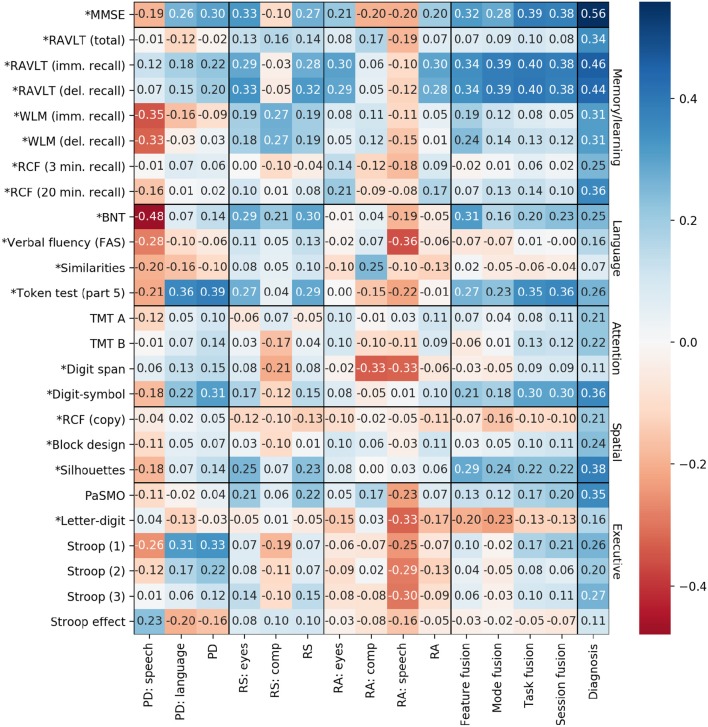
Spearman correlations between classifier predictions and neuropsychological test scores. PD, picture description; RS, reading silently; RA, reading aloud. Asterisks indicate the sign has been flipped so that all test scores correlate positively with diagnosis.

Spearman correlations are computed between the average probability scores produced by the LR classifier across the cross-validation folds, and the test scores. We also compute the correlation between each of the test scores and the actual diagnosis, to determine which tests are in fact associated with MCI status. In the classifier scores, a higher value always indicates a higher probability of MCI, but in many of the neuropsychological tests, a higher score indicates a lower probability of MCI (e.g., a higher MMSE score is associated with a lower probability of MCI). Therefore, to aid in interpretation, we adjust the sign of the correlations involving such test scores so that they are always positively correlated with MCI. The correlation coefficients are given in Figure 6, and the tests for which the signs were reversed are indicated with an asterisk. In our discussion, we refer to the conventional thresholds for correlation coefficients; namely, that values >0.5 indicate a strong correlation, values >0.3 indicate a moderate correlation, and values >0.1 indicate a weak correlation (Hemphill, 2003).

In general, the correlations between test scores and classifier predictions are not particularly high. Some correlations are even negative, suggesting that the classifier is making predictions that contradict the neuropsychological tests. We now examine each of our hypotheses from section 2.4.

Contrary to our expectation, the predictions based on speech features in particular tend to be negatively correlated with test scores, including those with a time component (PaSMO, TMT A and B, verbal fluency, block design, digit-symbol, and the Stroop measures).

Examining the correlations with the language mode, the only correlation that exceeds 0.3 is with the Token Test (Part 5). The language mode predictions are weakly positively correlated with BNT and weakly negatively correlated with the Letter Fluency test.

With respect to the higher level predictions, we do see an increasing correlation with MMSE at the level of task and session fusion predictions, as hypothesized. However, the task- and session-level scores are actually more highly correlated with RAVLT immediate and delayed recall than with MMSE, suggesting that the classifier predictions based on this particular set of tasks are more associated with learning/memory ability than overall cognitive ability. Specifically, scores from both of the reading tasks are correlated with RAVLT immediate and delayed recall, which is in line with the findings of Schmitter-Edgecombe and Creamer (2010), and confirms the intuition that the reading tasks in our protocol share similarities with existing standardized recall tasks.

### 4.5. Combining Predictions From Linguistic and Neuropsychological Data

The low correlations in the previous section would seem to suggest that the language tasks and the neuropsychological tests are capturing complementary information. Thus, it is reasonable to think that by combining these information sources, we could achieve even better classification performance. To investigate this idea, we combined the probability score output from the neuropsychological classifier with the probability scores from the picture description, reading aloud, and reading silently classifiers (this corresponds to the “task fusion” paradigm, but with four votes now instead of three). Fusing the information in this way led to an increase in AUC from 0.88 to 0.90 for both SVM and LR.

## 5. Discussion

### 5.1. Multimodal MCI Classification

Our classification results indicate that combining information from multiple sources leads to better results, and that the cascaded approach is superior to the early fusion baseline. Given the small size of our dataset, this may be partly due to the difficulty in training a classifier when the feature set size is larger than the number of training samples, which is the case in early fusion. The cascaded approach also permitted probability estimates to be obtained at the level of each mode and task, rather than a single, session-level probability. Our best overall result was obtained in the task fusion configuration, with an AUC of 0.88 and an accuracy of 0.83; while it is difficult to draw exact comparisons across different cohorts, this compares favorable with previous language-based machine learning studies on detecting MCI, which reported AUC = 0.732 (when excluding neuropsychological features) (Roark et al., 2011), AUC = 0.724 (Toth et al., 2018), AUC = 0.758 (König et al., 2018a), AUC = 0.796 (Asgari et al., 2017), and AUC = 0.82 (Lehr et al., 2013; Prud'hommeaux and Roark, 2015). However, it does not exceed the accuracy of 0.86 reported by König et al. (2018b) and Gosztolya et al. (2019) (AUC is not given in these cases). Nonetheless, we consider it a promising result compared to the alternative approaches in the literature.

Previous classification studies using the picture description task have combined acoustic and linguistic features (Roark et al., 2011; Fraser et al., 2016; Gosztolya et al., 2019). Here, we found that the linguistic features led to much better results than the speech-based features (AUC = 0.70–0.73, compared to 0.31–0.40). One explanation for this may be that linguistic features capture cognitive deficits more directly—for example, features, such as word frequency and content density are directly linked to semantic processing, which is known to deteriorate in the early stages of cognitive impairment (Barbeau et al., 2012; De Marco et al., 2017; Gosztolya et al., 2019). This finding is also consistent with the conclusion of Mueller et al. (2017), based on manual analysis of a much larger, longitudinal sample of MCI patients, that “features of connected language are associated with very early, sub-clinical declines in memory in late-middle age.” Interestingly, the speech features were more predictive in the classifier trained on the reading aloud task. This may be partially due to the fact that the reading task is more constrained in length and content (each participant reads the same text), and thus the samples are easier to compare from an acoustic perspective. However, as we will elaborate below, slowness on the reading task may also reflect a deliberate strategy by MCI participants to improve performance on the subsequent comprehension task, rather than any actual impairment in reading ability.

In the previous work on reading in MCI, reductions in recall and comprehension were reported (Hudon et al., 2006; Tsantali et al., 2013; Segkouli et al., 2016), and attributed to difficulties in episodic memory and the encoding of new information (Belleville et al., 2008). Our results are somewhat ambiguous on this point, as the classifiers trained on comprehension features achieved a maximum AUC of 0.60 on the silent reading task, and performed worse than random on the verbal reading task. In fact, we note that there was no significant difference in comprehension accuracy between the MCI and HC groups on either reading task, although MCI participants tended to take longer to answer the questions. Taking into account the multiple-choice nature of the comprehension questions, similar results have been reported in imaging studies: MCI patients showed no difference relative to controls on recognition performance, but with different underlying activation patterns in the prefrontal cortex, suggesting the potential contribution of compensatory activation (Clément et al., 2010).

Out of all the modes and tasks, eye-tracking during silent reading had the most predictive power. One interesting result is how much better the results on the *silent* reading task were compared to the *verbal* reading task. Our proposed explanation for this relates partly to our experimental protocol. In the silent reading task, participants were instructed to read the text, and tell the examiner when they were finished. Only when they indicated that they were finished did the comprehension questions appear. This allowed time, if necessary, to go back and re-read any portions of the text which were unclear or contained details to be remembered. This re-reading behavior is captured in the eye-movements. In contrast, in the reading aloud task, the participants simply read the text out loud, and when they reached the end, the examiner switched to the next screen, which displayed the comprehension questions. Thus, in the reading aloud task, readers were constrained to keep reading forward through the text as they read, and then were not given the opportunity to employ a re-reading at the end. Therefore, there were fewer differences in the eye-movement patterns between the MCI and control participants.

Both re-reading (as captured in the eye-tracking mode) and taking a longer time to consider the questions (as captured in the comprehension mode) may represent deliberate, and apparently effective, strategies by the MCI participants to achieve recall performance on par with the healthy controls. Given the fact that in the earliest stages of cognitive decline, performance on many tasks will be at ceiling—especially among a highly-educated cohort—these results suggest that in general there may be more value in focusing on a process-oriented approach to automated assessment (that is, observing the process by which a participant arrives at a result) rather than simply considering the final task performance.

### 5.2. Correlation Analysis

In general, we cannot draw a strong link between the probability scores output by the classifier and the neuropsychological test scores. Being able to do so would aid interpretability, as we could place the predictions in the familiar context of standardized test results. However, as we have discussed previously, producing narrative speech and reading a paragraph of text are complex cognitive tasks, and so a lack of clear correlation with more targeted cognitive tasks is perhaps unsurprising. Mueller et al. (2017) reached a similar conclusion after finding only weak correlations between cognitive test scores and performance on the Cookie Theft task, noting that poor narrative speech performance can be due to a number of factors other than impairment specifically relating to language, including memory deficits. Furthermore, we emphasize that the task of the classifiers is only to predict MCI status, and thus features which are highly correlated with specific test scores are not necessarily given high weights in the model, unless they also correlate with diagnosis. Given this, one possible avenue for future research could be to build regression models from the same features, with the specific objective of predicting scores on various neuropsychological tests, as an intermediate step toward the final classification.

### 5.3. Limitations and Future Work

One limitation of this work is the small sample size, which is unfortunately typical of many such studies due to the effort and expense of recruiting and assessing patients. We have attempted to mitigate the issue of small data by using simple classifiers, minimal optimization, and leave-pair-out cross-validation in order to avoid potential overfitting. However, future work should attempt to replicate these results in a bigger dataset with a wider range of educational and cultural backgrounds.

MCI is a heterogeneous condition with varying etiologies, and attempts to further characterize the cognitive impairments in MCI have resulted in four clinical subtypes (Gauthier et al., 2006). The participants in our study were not subtyped as part of the diagnostic process, and thus we have a mixed MCI group. While this can certainly lead to difficulties in machine learning classification (as discussed in section 4.3), we also observed that the false negatives produced by our multimodal classifier each came from a different subtype (with one unknown), suggesting that the classifier is generally able to handle the multiple subtypes together. However, given a larger dataset, future work should take into account these heterogeneities in MCI presentation.

To achieve our goal of a fully-automated assessment platform, there are many challenges still to address. Here, for example, we relied on manual transcription of the Cookie Theft task, and some features were extracted automatically and then corrected by hand. The benefit to this approach is that we have high confidence in the quality of our data. However, future work will examine the use of automatic speech recognition and other technologies to fully automate the processing pipeline. Additionally, this work has demonstrated the predictive power of eye-tracking for MCI detection, but we acknowledge that the equipment needed for this analysis is not readily available in most clinical settings. Again, future work will explore the use of emerging technologies for tracking eye movements with web-cams and other portable devices (Papoutsaki et al., 2016; Huang et al., 2017).

Previous research has reported mixed results on whether the Cookie Theft picture description task is sensitive to very early cognitive decline. In this study, we achieved moderate results with this task, but future work will also include additional language tasks which have been shown to detect MCI. Specifically, we are currently collecting data from the same cohort of participants using a semantic verbal fluency task (animals), a trip-planning task, and a dialogue-based map task. Our participants will also repeat the tasks described here, which will allow a longitudinal analysis of language changes in MCI and healthy aging. All participants will also return to the memory clinic for a follow-up GDS assessment, which will provide valuable information on disease progression and possible conversion to dementia. Finally, in future work we plan to incorporate MRI images and neurochemical biomarkers that were collected as part of the Gothenburg MCI study, to better understand the relationship between structural, chemical, and behavioral changes in MCI.

## 6. Conclusion

There has been growing interest in the possibility of using machine learning to detect early cognitive decline from speech and language. Speaking and reading are naturalistic tasks that can be easily repeated, but the manual analysis of such data can be time-consuming and expensive. Automated feature extraction and classification may aid in sensitive, longitudinal monitoring of cognitive function. Previous work has generally focused on a single language task; here, we present a framework for combining the data from several tasks, each represented via multiple modalities, to improve the discriminative performance of the classifier. We demonstrate that using a cascaded approach leads to the best result, and outperforms a classifier trained on test scores from a neuropsychological battery, as well as the more-commonly used early fusion method of multimodal learning. We have argued that the cascaded approach also improves *post-hoc* interpretability, as the probability estimates according to each mode and task can be observed, along with the final classification.

We emphasize that while the current analysis focused on language-based tasks, this architecture could be extended to incorporate data from other tasks and modalities, such as cognitive test scores, neuro-imaging, cerebrospinal fluid biomarkers, DNA information, and so on. Furthermore, while we limited our analysis to simple classifiers (LR and SVM), there is no reason why each component classifier could not be more complex, assuming there is enough data to support robust training.

Further work will be required to validate this methodology for other languages and a wider range of demographic groups, and some of the steps involved (e.g., eye-tracking) are not likely to be clinically feasible in their current state. Nonetheless, this preliminary result illustrates the potential sensitivity of language production and comprehension for detecting mild cognitive decline.

## Data Availability

The datasets for this manuscript are not publicly available because they contain sensitive and personally-identifying information. Requests to access the datasets should be directed to Dimitrios Kokkinakis, dimitrios.kokkinakis@svenska.gu.se.

## Ethics Statement

This study was carried out in accordance with the recommendations of the Regional Ethical Review Board of Gothenburg, Sweden with written informed consent from all subjects in accordance with the Declaration of Helsinki. The participants in this study were recruited from the Gothenburg MCI Study, which was approved with reference number: L091–99, 1999; T479–11, 2011. The protocol for the currently described study was approved with reference number 206–16, 2016.

## Author Contributions

KL and DK designed the study protocol and collected the data. KF, KL, and DK contributed to the feature extraction pipeline. ME and FÖ provided the neuropsychological scores and contributed to the correlation analysis and clinical interpretation. KF designed and implemented the classification experiments, conducted the analysis, and wrote the first draft of the paper. All authors contributed to manuscript revision, read, and approved the submitted version.

### Conflict of Interest Statement

KF has a financial interest in WinterLight Labs Incorporated, a company that commercializes the automated analysis of spontaneous speech. WinterLight Labs provided no influence over this work, nor has WinterLight Labs received any material benefit from this work. The remaining authors declare that the research was conducted in the absence of any commercial or financial relationships that could be construed as a potential conflict of interest.
